# Artificial Intelligence as a Clinical Co-Pilot in Reminiscence Therapy: A Nurse-Led Model for Super-Aged Societies

**DOI:** 10.31662/jmaj.2025-0552

**Published:** 2026-03-27

**Authors:** Kazumi Kubota, Yukiko Matsumura

**Affiliations:** 1 Research Organization, Shimonoseki City University, Shimonoseki, Japan; 2 Department of Healthcare Information Management, The University of Tokyo Hospital, Tokyo, Japan; 3 Health and Global Policy Institute, Tokyo, Japan; 4 Department of Nursing, Faculty of Nursing, Shimonoseki City University, Yamaguchi, Japan

**Keywords:** artificial intelligence, reminiscence therapy, dementia care, human-in-the-loop, geriatric nursing

## Abstract

Japan’s demographic landscape, characterized as a super-aged society, presents an urgent need for scalable psychosocial interventions for dementia and cognitive decline. Reminiscence therapy is an evidence-based non-pharmacological intervention; however, its implementation is often limited by workforce shortages and time constraints. This opinion paper proposes a paradigm shift: utilizing Artificial Intelligence (AI) not as a replacement for caregivers, but as a “clinical co-pilot” under nursing leadership. While generative AI and robotics offer transformative potential to personalize content and extend therapeutic reach, they also introduce risks such as algorithmic bias and “hallucinations” that could distress patients. We argue that successful integration requires a “Human-in-the-Loop” framework in which AI handles data synthesis and engagement monitoring, while healthcare professionals maintain oversight of clinical validity and emotional safety. This approach aims to harmonize technological efficiency with person-centered care, offering a sustainable model for geriatric medicine.

## Introduction

### The scalability crisis and the artificial intelligence (AI) co-pilot solution

Japan faces a critical healthcare paradox: the prevalence of dementia is rising alongside a shrinking workforce. While pharmacological treatments for dementia have limitations, non-pharmacological interventions such as Reminiscence Therapy (RT) are recommended by clinical guidelines to improve quality of life and cognition ^(1)^. However, traditional RT is labor-intensive, requiring skilled facilitators to curate personalized triggers―such as photos, music, and stories―and guide conversations. In many clinical settings, this “dosage” of therapy is insufficient due to resource constraints. Recent advances in AI offer a potential solution to this scalability crisis; however, the application of these technologies requires rigorous validation and careful consideration of their limitations, mirroring the scrutiny currently applied to AI in diagnostic fields.

To address these challenges, we propose defining AI in this context as a “Clinical Co-Pilot.” Unlike autonomous systems, a co-pilot supports the primary operator―in this case, the nurse or therapist. In practice, generative AI can instantly curate “Digital Life Storybooks” by analyzing patient history, significantly reducing the preparation burden on staff ^(2)^. Beyond content creation, AI-driven sensors can analyze facial expressions and voice tone to assess patient engagement in real-time, providing objective data to the medical team. Furthermore, AI-enabled interfaces may support delivery in home-care or resource-limited settings; however, the central clinical question is not the device itself but how content is generated, verified, and governed before it reaches a cognitively vulnerable patient.

**Figure 1.**
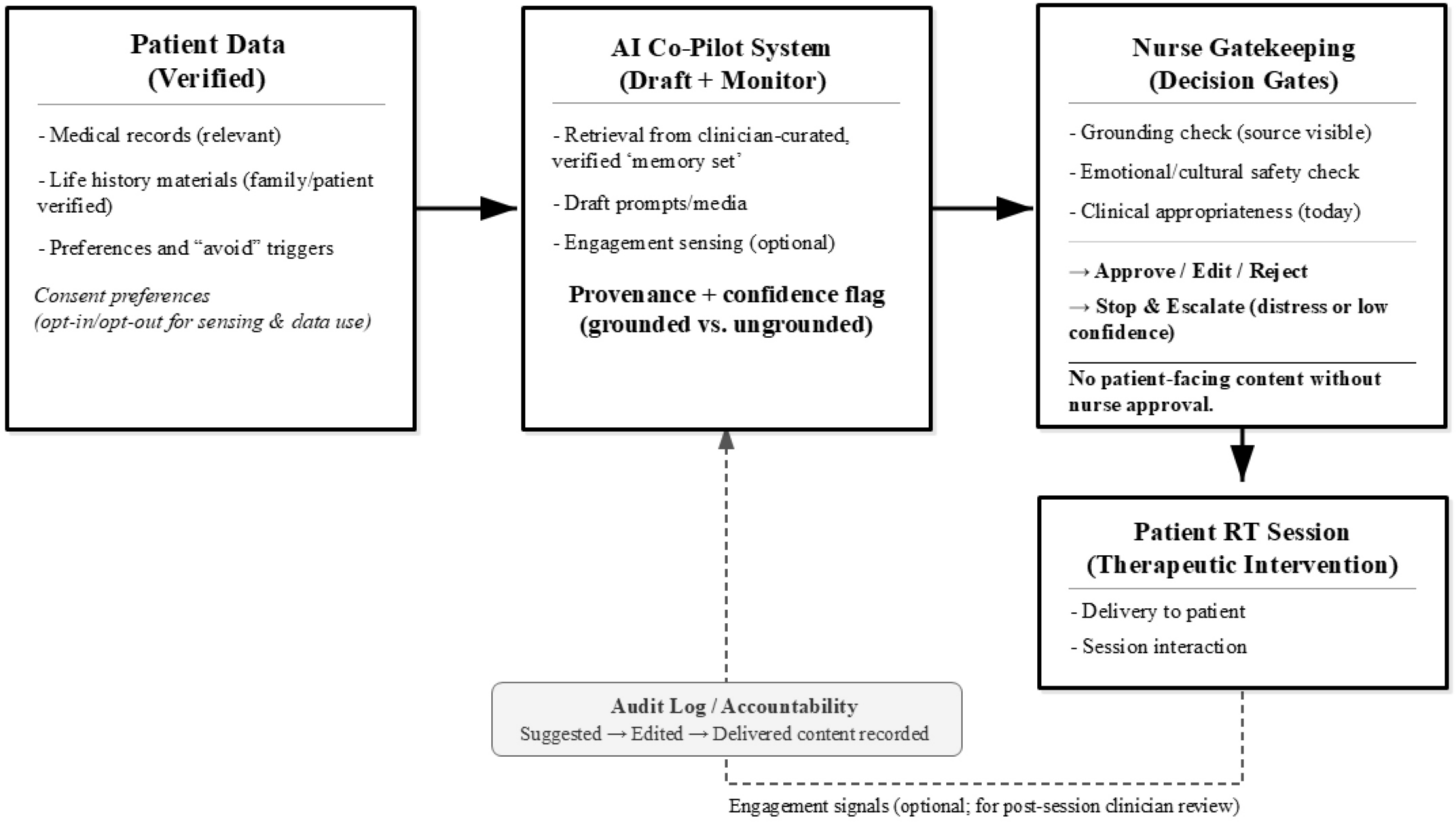
AI co-pilot system workflow for nurse-led reminiscence therapy. Verified patient data are curated into a clinician-approved “memory set” used for grounded drafting. Nurses apply decision gates (grounding, emotional/cultural safety, and clinical appropriateness) and determine whether to approve, edit, reject, or stop and escalate (distress or low confidence); no patient-facing content is delivered without nurse approval. Engagement signals are optional and intended for post-session clinician review. An audit log records suggested, edited, and delivered content to support accountability and incident review. AI: artificial intelligence.

While these capabilities are promising, the “black box” nature of algorithms remains a critical concern. In the context of RT, generative AI could “hallucinate” incorrect historical details or produce culturally inappropriate content, causing confusion or distress in a cognitively vulnerable patient. Therefore, a nurse-led Human-in-the-Loop architecture is essential. Here, “clinical decision augmentation” means that AI supports preparation and monitoring, while nurses verify outputs and calibrate risk rather than delegating decisions to autonomous AI. Drawing on Park’s “Sweet Spot” logic, oversight intensity increases as patient vulnerability, content sensitivity, or AI uncertainty increases―operationalized as explicit decision gates (approve/edit/reject) and a stop-escalation pathway (Figure 1) ^(3), (4)^.

Although Japan is used here as a super-aged exemplar, the core design principle―nurse-led Human-in-the-Loop governance with proportional oversight―can translate to other healthcare systems facing workforce constraints. Local adaptation will be needed for scope-of-practice/team roles, data protection, language/cultural grounding, and reimbursement/workflows.

### Clinical benefits of AI-assisted RT

#### Enhanced personalization and engagement

The therapeutic value of RT lies in connecting with an individual’s life history and preferences ^(1)^. AI may reduce staff preparation time by helping organize a verified “memory set” and suggesting session prompts; however, therapeutic value depends on facilitation and verification rather than automation. A practical example of technology-mediated reminiscence work is the use of multimedia digital life storybooks, which can support personalized triggering of positive memories and emotions when materials are curated and verified ^(2)^. Subramaniam and Woods reported that digital life storybooks were feasible and well-received in care homes, with signals of improvement in autobiographical memory and quality of life, underscoring the importance of curated, person-specific content ^(2)^.

#### Cognitive stimulation and therapeutic outcomes

Beyond simple recall, AI may support structured prompts and help clinicians track engagement patterns to refine facilitation over time. However, for cognitively vulnerable populations, a key risk is over-attributing benefit to an interface while overlooking content accuracy and emotional safety. This is why the proposed model centers on nurse gatekeeping of patient-facing content and explicit stop-escalation rules (distress or low confidence), rather than on any specific device or platform.

### Extended reach and accessibility

AI technologies may expand access to reminiscence-oriented interventions beyond traditional clinical settings, including remote or resource-limited contexts. However, scalability should not come at the expense of consent, verification, and clear escalation pathways. The model, therefore, prioritizes a nurse-led workflow that preserves accountability even when delivery formats vary.

#### Clinical implementation challenges and risk management

When AI-assisted RT may fail or be inappropriate

AI-assisted reminiscence may be inappropriate when there is a high risk of distress (e.g., trauma- or grief-related triggers), when verified life-history materials are insufficient for grounding, or when the system signals low confidence. In such cases, the default should be to pause AI-generated content, prioritize human-led therapeutic judgment, and activate a clear stop-escalation pathway.

Scenarios warranting avoidance, deferral, or escalation include: (1) advanced/severe dementia with limited ability to engage meaningfully or provide assent; (2) unresolved trauma, complicated grief, or repeatedly distressing reminiscence themes; (3) lack of verifiable life-history data to construct a clinician-approved, grounded “memory set”; (4) acute clinical instability “today” (e.g., delirium, severe agitation, or intercurrent illness) that changes risk calibration; and (5) repeated ungrounded outputs or low-confidence flags despite nurse editing.

Over-dependence on technology may also unintentionally reduce human contact; therefore, AI should remain optional, reversible, and explicitly framed as support for (not a substitute for) therapeutic relationships.

### Digital literacy and usability barriers

A fundamental challenge in AI implementation involves varying levels of digital literacy among both older adults and healthcare providers. In practice, even well-designed interfaces may still require ongoing technical support for some users. This necessitates comprehensive training programs for healthcare professionals and dedicated support systems for older adults and their families. User-centered design principles must guide AI development, incorporating voice commands, simplified controls, and culturally appropriate interfaces. Healthcare organizations must invest in “digital coach” programs and provide ongoing technical support to ensure successful adoption across diverse populations.

### Ethical and privacy considerations

RT involves deeply personal and often sensitive information, raising significant privacy and security concerns. AI systems must implement robust data encryption, secure storage protocols, and transparent consent processes that respect patient autonomy while enabling therapeutic benefit. The risk of AI-generated errors or culturally inappropriate content requires continuous human oversight and the ability to correct or override AI recommendations. Cultural sensitivity emerges as a critical factor, particularly in diverse societies such as Japan. Reminiscence content is inherently culture-bound, tied to specific languages, customs, and historical contexts. AI systems must be trained on diverse datasets and designed with cultural adaptability to avoid generic content that may not resonate with specific populations.

### Preserving the human element

The integration of AI must not compromise the empathetic, intuitive judgment that skilled nurses and therapists bring to RT. AI should augment human capabilities rather than replace them, serving as a sophisticated tool that enhances the therapeutic relationship. Clear protocols must ensure that human professionals maintain oversight of AI-driven sessions, particularly when emotional distress arises.

#### A proposed framework for responsible implementation

Nurse-Led Integration

Nurses, with their holistic understanding of patient needs, ethical grounding, and direct patient contact, are uniquely positioned to lead AI integration in RT. Their clinical judgment, proximity to patients, and commitment to person-centered care make them ideal stewards of technology implementation. Professional development programs must prepare nurses to work effectively with AI tools while maintaining their essential role in therapeutic relationships.

### Evidence-based deployment

Healthcare organizations should adopt a phased, evidence-based approach to AI integration. Initial implementation should focus on pilot programs in controlled settings with robust evaluation frameworks measuring clinical outcomes, patient experience metrics, and cost-effectiveness. These pilots should include diverse patient populations to ensure cultural appropriateness and identify potential disparities in access or effectiveness.

### Ethical and regulatory frameworks

Institutional policies must govern AI use in RT, including protocols for human supervision, data privacy protection, and intervention when necessary. Regulatory frameworks should evolve to address the unique challenges of AI in psychosocial interventions, establishing standards for content accuracy, privacy protection, and clinical oversight requirements. We therefore specify minimum governance requirements and evaluation metrics. At minimum, safe deployment requires (1) consent preferences, including opt-in/opt-out options for sensing and data use; (2) provenance/grounding checks with visible sources; (3) “confirm-before-assert” defaults and hard stops when outputs are ungrounded or low confidence; and (4) audit logging of suggested, edited, and delivered content to support accountability and incident review.

In practice, these safeguards can be integrated into routine RT preparation and nurse approval, with provenance/confidence levels shown at sign-off and audit logs captured automatically to minimize added burden; sensing is optional for post-session review.

Bias and cultural inappropriateness should be addressed through curated memory sets, culturally safe prompting, and structured clinician review. For pilots, a minimal evaluation set should include safety (distress/adverse events), effectiveness (engagement/therapeutic goals), and equity (performance across subgroups) ^(5)^.

## Conclusions

### Toward evidence-based implementation

The integration of AI into RT represents a paradigm shift that could fundamentally improve care for aging populations. However, realizing this potential is not merely a technological opportunity but a clinical imperative that requires rigorous validation. To move forward, clinical trials are needed to compare AI-assisted RT with traditional methods, focusing not only on cognitive scores but also on safety, algorithmic transparency, and cost-effectiveness. By establishing a nurse-led Human-in-the-Loop framework (Figure 1) in which AI supports the “logistics” of preparation while humans safeguard the “meaning,” this model can guide cautious translation into practice. However, wide adoption should follow phased pilots with prespecified safety stop criteria, transparent governance, and evaluation across safety, effectiveness, and equity endpoints.

## Article Information

### Author Contributions

Conceptualization: Yukiko Matsumura, Kazumi Kubota. Literature review and selection of supporting evidence: Kazumi Kubota, Yukiko Matsumura. Drafting the original manuscript: Kazumi Kubota. Revision of the manuscript in response to peer review (theoretical framework integration; governance/metrics clarification; and restructuring for balanced critical tone): Kazumi Kubota, Yukiko Matsumura. Development and revision of Figure 1: Kazumi Kubota, Yukiko Matsumura. Critical revision for important intellectual content: Yukiko Matsumura, Kazumi Kubota. Supervision: Kazumi Kubota. Both authors contributed equally and share first authorship.

### Conflicts of Interest

None

### Ethics Approval and Consent to Participate

Not applicable. This manuscript is an opinion article and does not involve new clinical studies of human participants or animals.

### Statement on Artificial Intelligence Use

In the preparation of this manuscript, the authors utilized an artificial intelligence-based language model (ChatGPT; OpenAI, San Francisco, CA, USA) to refine English phrasing and ensure structural coherence. The core concepts, clinical opinions, and final review of the content were performed entirely by the human authors, who take full responsibility for the integrity of the work.

## References

[1] Woods B, O’Philbin L, Farrell EM, et al. Reminiscence therapy for dementia. Cochrane Database Syst Rev. 2018;3(3):CD001120. 29493789 10.1002/14651858.CD001120.pub3 PMC6494367

[2] Subramaniam P, Woods B. Digital life storybooks for people with dementia living in care homes: an evaluation. Clin Interv Aging. 2016;11:1263-76. 27698556 10.2147/CIA.S111097 PMC5034922

[3] Park CS. Optimizing staffing, quality, and cost in home healthcare nursing: theory synthesis. J Adv Nurs. 2017;73(8):1838-47. 28464325 10.1111/jan.13284

[4] Park CS. “More is not always better”: Park’s sweet spot theory-driven implementation strategy for viable optimal safe nurse staffing policy in practice. Int Nurs Rev. 2023;70(2):149-59. 35817044 10.1111/inr.12785

[5] Park CS. Ethical artificial intelligence in nursing workforce management and policymaking: bridging philosophy and practice. J Nurs Manag. 2025;2025:7954013. 40236787 10.1155/jonm/7954013 PMC11999746

